# Deacetylation of Chitosan: Material Characterization and *in vitro* Evaluation via Albumin Adsorption and Pre-Osteoblastic Cell Cultures

**DOI:** 10.3390/ma4081399

**Published:** 2011-08-12

**Authors:** Youling Yuan, Betsy M. Chesnutt, Warren O. Haggard, Joel D. Bumgardner

**Affiliations:** Department of Biomedical Engineering, University of Memphis, Memphis, TN 38152, USA; E-Mails: betsy.chesnutt@gmail.com (B.M.C.); whaggrd1@memphis.edu (W.O.H.); jbmgrdnr@memphis.edu (J.D.B.)

**Keywords:** chitosan, molecular weight, degree of deacetylation, material characterization, cell attachment and proliferation

## Abstract

Degree of deacetylation (DDA) and molecular weight (MW) of chitosans are important to their physical and biological properties. In this study, two chitosans, HS (DDA = 73.3%) and AT (DDA = 76.8%), were deacetylated with 45% sodium hydroxide under nitrogen atmosphere at 80 °C or 90 °C for up to 120 min, to obtain two series of chitosans. The polymers produced were characterized for MW by gel permeation chromatography, DDA by titration and UV-vis methods, and crystallinity, hydrophilicity and thermal stability by X-ray diffraction, water contact angle and differential scanning calorimetry respectively. Films, made by solution casting in dilute acetic acid at ambient conditions, were evaluated for biological activity by albumin adsorption and the attachment and growth of a pre-osteoblast cell line. Chitosans with between 80–93% DDA’s (based on titration) were reproducibly obtained. Even though deacetylation under nitrogen was supposed to limit chain degradation during decetylation, MW decreased (by maximum of 37.4% of HS and 63.0% for AT) with increasing deacetylation reaction time and temperature. Crystallinity and decomposition temperature increased and water contact angles decreased with processing to increase DDA. Significantly less albumin was absorbed on films made with 93% DDA chitosans as compared with the original materials and the AT chitosans absorbed less than the HS chitosans. The cells on higher DDA chitosan films grew faster than those on lower DDA films. In conclusion, processing conditions increased DDA and influenced physicochemical and biological properties. However, additional studies are needed to unambiguously determine the influence of DDA or MW on *in vitro* and *in vivo* performance of chitosan materials for bone/implant applications.

## 1. Introduction

Chitosan is a non-toxic, biocompatible, and biodegradable polymer that exhibits promise in a wide range of biomedical applications including wound dressings, tissue engineering, implant coatings and therapeutic agent delivery systems [1,2,3,4,5]. Chitosan degree of deacetylation (DDA) has often been cited as an important parameter that determines many physiochemical and biological properties of chitosans such as crystallinity, hydrophilicity, degradataion and cell response [6,7,8,9,10,11]. For example, Freier *et al*. [7] found that prolonged degradation times and enhanced cell adhesion can be achieved using chitosan with a DDA close to 0% or 100% while chitosans with intermediate DDAs exhibit rapid degradation rates, but at the cost of limited cell adhesion. Hidaka *et al*. [10] reported that chitosan membranes between 65 and 80% DDA elicited marked inflammatory reactions that subsided in time with degradation of the films, granulation tissue formation, and osteogensis while membranes made of 94% DDA chitosan showed minimal degradation, mild inflammation and minimal osteogenesis.

DDA of chitosan is generally controlled by processing of the native polymer with alkali, and with increasing time and temperature to obtain the highest DDA (>90) materials [3,5,8,11,12]. The molecular weight (MW) of chitosan is dependent on the initial source material (shrimp, crab, fungi, *etc*.) and can decrease with processing to increase DDA [3,5,8,11,12]. MW has been shown to be an important factor in chitosan properties such as crystallinity, degradation, tensile strengths and moisture content [13,14,15,16,17,18]. For example, Nuthanid *et al*. reported that tensile strength and moisture adsorption of chitosans with high MW_v_’s (600–1000 kDa) were significantly greater than those with similar DDA’s but lower MW_v_’s (50–60 kDa) [18]. Further, Xu and Du reported that increasing chitosan MW (10–120 kDa) increased protein encapsulation efficiencies and reduced release kinetics. However, there has been little data on the role MW on biological performance. Hamilton *et al*. [19] evaluated the growth of osteoblasts on seven different chitosans (76–96 DDA and MW_v_ = 2400–8200 kDa) and noted there was no relationship between DDA, MW_v_ or growth of cells. Similarly, there were no correlations between DDA, MW or growth of keratinocytes on five different chitosans (53–97 DDA, MW_v_ = 735–1180 kDa) [20]. The most likely reason for the lack of correlation in these studies is due to differences in the initial source materials and process conditions for the different production lots.

Although serial DDA or MW chitosans have been prepared, systemic research on the effects of DDA or MW on chitosan properties after deacetylation has rarely been reported [14,15,21,22]. Huang *et al*. noted that the uptake of chitosan nanoparticles by cultured fibroblasts was mainly attributed to DDA but that MW also played a role [22]. Recently, Cao *et al*. [11] prepared six kinds of chitosan films with similar MW but various DDAs. They measured film crystallinities, swelling and mechanical properties, protein adsorption, and primary rat Schwann cell spreading and proliferation on the films. They found that DDA had a marked effect on the physicochemical properties and Schwann cell affinity of chitosan films. Higher DDA chitosan films showed a greater crystallinity, a higher elastic modulus and tensile strength and a lower swelling index than those with lower DDA. Even though this study provides insight into the true effects of DDA on properties and performance, these results are limited only to Schwann cells. Since chitosans have shown much promise in bone tissue engineering and implant applications [1,3,4,10], properties and performance characteristics need to be developed for bone. In this study work, a comprehensive study was undertaken to compare processing conditions on the physiochemical and biological properties of chitosan materials from two sources. Two low DDA chitosans were deacetylated under an inert atmosphere with hot sodium hydroxide with the aim of producing two series of chitosans with different DDAs but similar MWs. Three methods were used to determine chitosan DDA. The effect of DDA on the physical properties of residual protein, ash content, crystallinity, contact angle and thermal stability and biological properties of protein absorption and bone cell attachment and proliferation were studied.

## 2. Materials and Methods

### 2.1. Sodium Hydroxide Deacetylation of Chitosan [14]

Two chitosans with low DDA from different manufacturers were obtained (Table 1). Both chitosans were derived from shrimp sources, but from different locations (NW Pacific Ocean *vs.* Gulf of Mexico). Chitosan powders were added to a 45% sodium hydroxide solution in a 1:10 (W/V) ratio in a reaction vessel following Zhang’s method [14] and the reaction was under nitrogen atmosphere. The powders were subjected to one of the following reaction conditions: (1) 80 °C for 20 min; (2) 80 °C for 60 min; (3) 90 °C for 60 min; or (4) two 60 min treatments at 90 °C (Table 2 and Table 3). After reacting, the product was filtered and washed with distilled water until a neutral pH was obtained. The resulting products were dried in an oven for 24 h at 40 °C, then in a vacuum oven for 24 h at 40 °C.

**Table 1:** Chitosan properties.

Chitosan	Manufacturer	Source	DDA	Molecular weight ^c^ × 10^−4^
HS	Vanson HaloSource Redman, WA	Shrimp	78.7 ^a^	2.03 ± 0.05
AT	AgraTech International, Gulfport, MS	Shrimp	75.7 ^b^	3.16 ± 0.07

^a^ Provided by manufacturer; ^b^ Obtained by acid-base titration; ^c^ Determined by GPC.

**Table 2:** Chitosan deacetylation reaction under nitrogen atmosphere (batch 1 and 2).

Sample	Reaction temperature (°C)	Reaction time (min)	Batch 1		Batch 2	
			DDA (Acid-base)	MW (GPC) ×10^−4^	DDA (Acid-base)	MW (GPC) ×10^−4^
HS0	/	/	72.57 ± 0.18	2.03 ± 0.05	72.57 ± 0.18	2.03 ± 0.05
HS1	80	20	82.52 ± 0.07	1.25 ± 0.06	81.49 ± 0.38	1.19 ± 0.02
HS2	80	60	84.58 ± 0.34	1.16 ± 0.03	83.18 ± 0.17	1.12 ± 0.03
HS3	90	60	86.38 ± 0.19	1.02 ± 0.05	84.91 ± 0.13	1.01 ± 0.23
HS4	90	60 + 60	86.90 ± 0.06	0.83 ± 0.03	85.95 ± 0.09	0.76 ± 0.16
AT0	/	/	75.74 ± 1.27	3.16 ± 0.07	75.74 ± 1.27	3.16 ± 0.07
AT1	80	60	82.53 ± 1.43	2.18 ± 0.24	82.80 ± 0.07	2.10 ± 0.18
AT2	90	60	85.49 ± 0.11	2.11 ± 0.02	84.11 ± 1.76	2.08 ± 0.16
AT3	90	60 + 60	86.41 ± 0.26	1.84 ± 0.07	85.95 ± 0.49	1.99 ± 0.10

Statistics analysis of DDA and MW showed there was no difference for DDA and MW between the two batches.

**Table 3:** Chitosan characteristics of deacetylated products.

Sample	Reaction temperature (°C)	Reaction time (min)	Degree of deacetylation (%)			
			Titration (Acid-base)	Titration (Potentiometer)	UV-vis	MW (GPC) ×10^−4^
HS0	/	/	72.57 ± 0.18	73.31 ± 0.41	75.65 ± 0.69	2.03 ± 0.05
HS1	80	20	82.01 ± 0.62	80.18 ± 0.16	85.72 ± 0.40	1.22 ± 0.05
HS2	80	60	83.18 ± 0.80	83.12 ± 0.04	88.51 ± 0.57	1.14 ± 0.03
HS3	90	60	85.64 ± 0.82	89.47 ± 0.40	91.42 ± 0.62	1.02 ± 0.14
HS4	90	60 + 60	86.45 ± 0.55	92.91 ± 0.35	93.96 ± 0.04	0.75 ± 0.13
AT0	/	/	75.74 ± 1.27	76.77 ±0.05	85.38 ± 0.16	3.16 ± 0.07
AT1	80	60	82.67 ± 0.92	86.15 ± 0.36	96.47 ± 0.27	2.12 ± 0.18
AT2	90	60	84.81 ± 0.56	89.63 ± 0.6	97.73 ± 0.09	2.09 ± 0.12
AT3	90	60 + 60	86.68 ± 0.46	93.93 ± 1.17	99.68 ± 0.12	1.93 ± 0.12

Statistics analysis of DDA showed: For DDA: Method statistical groups (Tukey’s LSD test p < 0.05): there are differences due to method (p = 0.0131) and reaction time (0.0022). Acid-base and Potentiometer are not different and Potentiometer and UV/Vis are not different. Reaction time groups (by One Factor ANOVA and Tukey’s LSD test (p < 0.05)): For acid base: all groups are different; for Potentiometer: all groups are different; for UV-vis: all groups different except AT2 and AT3 are the same. Statistics analysis of MW showed significantly different samples for HS chitosan: HS0 *vs.* all others; HS4 *vs.* all others and HS1 *vs.* HS3; For AT chiotsan: AT0 *vs.* all others.

### 2.2. Preparation of Chitosan Films

1.0 g chitosan was dissolved in 99.0 mL 1 wt% acetic acid to get a 1 wt% solution. The solution was stirred for two h to allow the chitosan to dissolve completely, and then poured into a round Teflon dish. Films were dried at room temperature and then neutralized by quickly rinsing with 0.05 M NaOH, followed by rinsing in deionized water and finally re-dried at room temperature.

## 3. Chitosan Characterizations

### 3.1. Determination of Degree of Deacetylation (DDA)

#### 3.1.1. Acid-Base Titration [23]

Chitosan (0.3–0.5 g) was dissolved in 30 mL 0.1 M HCl at 20 ± 5 °C with stirring in a 250 mL flask and then two drops of methyl orange indicator was added. 0.1 M NaOH was used to titrate the solution. At the final point of titration, the color changes from pink to orange yellow. The method was augmented to include the use of a pH meter to make the final titration point determination more precise. To calculate water content, 0.5 g chitosan was heated at 105 °C until a constant weight was reached. Three parallel samples were used. The percent of free NH_2_ groups in chitosan was calculated as follows:

NH_2_% = [(C_1_V_1_ − C_2_V_2_) × 0.016] / [G (100 − W)] ×100 (1)

Free NH_2_% = NH_2_%/9.94% × 100%

Chitosan theoretic NH_2_ content % = (16/161) × 100% = 9.94%

C_1_: Concentration of HCl (M); C_2_: Concentration of NaOH (M); V_1_: the volume of HCl added (mL); V_2_: the volume of NaOH added by titration (mL); G: Sample weight (g); W: sample water content (%); 0.016: equal to NH_2_ content (g) in 1 mL of 1 M HCl.

#### 3.1.2. Potentiometer Titration [24]

Chitosan (0.5 grams) was dissolved in 20 mL of 0.3 N hydrochloric acid. After adding 400 mL of distilled water, this solution was titrated with a 1 N NaOH solution. A titration curve of pH *vs.* NaOH titration volume was generated. The curve’s inflection points were found for each indicated transition. The volume of NaOH at the each inflection point was applied to the equation:

NH_2_% = 16.1×(y − x)/M (2)

where M is the weight of chitosan used, x is the first inflection point on the graph of measured pH *vs.* titration volume, and y is the second inflection point.

#### 3.1.3. Ultraviolet-Vis Spectrophotometry [23,25]

N-acetylglucosamine was dissolved in 0.001 mol/L HCl to prepare 0.1 mg/mL standard solution. A series of 0.01, 0.02, 0.03, 0.04 and 0.05 mg/mL standard solutions was prepared from the 0.1 mg/mL standard solution and the absorbance of each solution at 199 nm was determined on a UV-vis Spectrophotometer (Agilent 8453 instrument) using 0.001 mol/L HCl as reference. A standard curve of concentration *vs.* absorbance was generated. Chitosan (10–20 mg) was dissolved in 10 mL 0.01 mol/L HCl in a 100 mL volumetric flask. After the chitosan was dissolved completely, the solution was diluted to 100 mL using de-ionized water. According to the standard curve, the concentration of acetyl can be determined by measuring the solution absorbance at 199 nm. DDA can be calculated according to the equation:

DDA=100% − C_1_/C (3)

where C_1_ is the acetyl concentration of sample and C is concentration of sample.

### 3.2. Ash Content [23]

Chitosan ash content was determined by combustion using a constant weight crucible. The crucible was repeatedly placed into an oven at 550 °C ± 20 °C for 30 min and then removed, cooling for 30 min in dessicator, and then weighed until a constant weight W_0_, ± 0.5 mg was obtained. Chitosan (2–5 g) was combusted in the constant weight crucible in an oven at 550 °C ± 20 °C for 3 h. The crucible was removed, cooled in a desiccator for 30 min, and re-weighed (W_1_). This heating and cooling process was repeated every 1.5 h until a constant weight was established (W_2_). The ash percentage was calculated by the equation:

(4) $$Ash\%=\frac{W_{2}-W_{0}}{W_{1}-W_{0}}\times 100$$

where W_0_ is the constant weight of crucible, W_1_ is the weight of sample and crucible, W_2_ is the weight of ash and crucible. The ash content was determined from two samples.

### 3.3. Protein Assay

Chitosan residual protein concentrations were determined by Bradford assay (Sigma-Aldrich Corp. St. Louis, MO, USA). Chitosan sample solution concentration was 10 mg/mL in 0.1 M acetic acid for the protein assay. The solutions were tested with the Bradford reagents according to the manufactures instructions and read at 595 nm (µQuant Universal Microplate Spectrophotometer; Bio-Tek Instruments, Inc., Winooski, VT, USA). The protein concentration was determined from five samples for each DDA chitosan. Phosphate buffered saline (PBS) and acetic acid (HAc) were used to prepare protein standard solutions respectively.

### 3.4. Gel permeation Chromatography (GPC)

The molecular weight of chitosan was measured by GPC. Pullulans with a molecular weight range from 1.05 × 10^5^ to 8.25 × 10^5^ (American Polymer Standards Corporation, Mentor, OH, USA) were used as standard samples to establish the universal calibration curve. The GPC equipment consists of Varion Prostar 350 RI Detector, 410 Autosampler, 210 Solvent Delivery, TSK-GEL G6000PWXL and GMPWXL Columns (Column temperature is 30 °C) and Galaxie Chromatography workstation software (v 1.8.501.1). The concentration of chitosan solution was 1 mg/mL in 0.2 M CH_3_COOH/0.1 M CH_3_COONa. Injection volume was 50 µL, flow rate was 0.5 mL/min, and continuous phase was 0.2 M CH_3_COOH/0.1 M CH_3_COONa. All the solvents and solutions were filtered through a 0.45 µm filter (Whatman Inc., Clifton, NJ, USA).

### 3.5. Differential Scanning Calorimetry (DSC)

Differential Scanning Calorimetry (DSC) measurements were carried out on a Netzsch DSC 200 (Germany). An accurately weighed 5–8 mg sample was placed in an aluminium cup and hermetically sealed. An empty cup was used as reference and runs were performed in duplicates. Samples were analyzed under continuous flow of dry nitrogen gas (10 mL/min) at a heating rate of 20 K/min from 20 to 450 °C.

### 3.6. X-Ray Diffraction (XRD)

Powders were further analyzed by X-ray diffraction to evaluate crystallinity [6,26]. X-ray diffraction spectra were collected on a Bruker D8 Advance X-ray diffractometer using CuKα radiation at 40 kV and 30 mA. The 2θ scan range was 4–40° with a step size of 0.1° and a time/step of 1 s. The crystallinity index, CrI, of the samples was calculated as:

CrI = (I_110_ − I_am_)/I_110_ (5)

where I_110_ is the maximum intensity of the (110) diffraction peak at 2θ = 20° and I_am_ is that of the amorphous diffraction signal at 2θ = 16°. Two samples of each chitosan powder were evaluated and the CrI was reported as the average (n = 2) ± standard deviation.

### 3.7. Contact Angle

Sessile drop air/water contact angle measurements were performed on chitosan films using a static contact angle goniometer (VCA Optima System, Advanced Surface Technology, MA, USA). The contact angle was determined from five samples for each DDA chitosan.

### 3.8. Protein Adsorption

Bovine albumin (pH 7.0) (MP Biomedicals, INC) was used for protein adsorption. A 0.9 cm diameter disk of each chitosan was punched from films and placed in a 48-well plate A 0.5 mL (per sample) of albumin [500 µg/mL in phosphate buffer saline (PBS)] was pipetted onto each test sample. Supernatant that contained non-adsorbed protein was removed after 30 min or 2 h. The films were washed using PBS and the wash solution was collected. A BCA assay was used to determine the concentrations of the supernatant containing non-adhered protein and the wash solution at 562 nm. The amount of protein adsorbed was determined by subtracting the concentrations of non-adhered protein plus the wash solution from the original protein concentration. Each type of chitosan was tested in triplicate for each time period.

### 3.9. Cell Attachment and Culture Test

Cell culture studies using human embryonic palatal mesenchyme cells (HEPM, CRL-1486, ATCC, Manassas, VA, USA), an osteoblast precursor cell line, were used to evaluate the cytocompatibility of the chitosan coatings. The cells were grown and maintained in 90% Dulbecco’s modified eagle medium (DEME, Invitrogen, Carlsbad, CA, USA) supplemented with 7% fetal bovine serum (FBS, Invitrogen) and 1% antibiotic/antimycotic (100U/mL penicillin, 100 µg/mL streptomycin, 0.25 µg/mL amphotericin (GIBCO BRL, Gaithersburg MD, USA) in an incubator at 37 °C with a 100% humidified 5% CO_2_ atmosphere. Gas sterilized chitosan films were placed in individual wells of 48 well plates. Cells were seeded at 1 × 10^5^ cells/cm^2^ for each specimen and tissue culture plates as a control. Attachment behavior of HEPM cells to chitosan film surfaces was evaluated after attached for 4 h. The cell media was collected along with two washes of PBS (phosphate buffered saline) to count unattached cells using a coulter (Backman Coulter Z2, Fullerton, CA, USA). The percent cell attachment was calculated as [%Att = (cells seeded − unattached cells)/cells seeded × 100%]. Cells were cultured for 7 days with media changes every three days. The growth of the cells was evaluated at 1-, 4- and 7-day intervals by counting cells. At each time point, media was removed and the specimen was washed three times with PBS, and then put in 0.2 mL of trypsin in each specimen and counted the cells. Four specimens of type of films were used for each time period.

### 3.10. Statistical Analyses

Analysis of variance (ANOVA) was performed to determine statistical differences. If significant differences were indicated (p < 0.05), Student-Newman-Keuls and Tukeys tests were performed. Statistical differences were declared at p < 0.05.

## 4. Results and Discussion

Two low DDA chitosans from different manufacturers were de-acetylated using 45% NaOH with different reaction temperatures and times to produce two series of chitosan materials with different DDA. Reactions were performed under nitrogen to minimize decreases in MW of the chitosan polymer, in order to evaluate the effects of DDA on chitosan physical and biological properties without the confounding effects of changing MW. The results of the de-acetylation of two batches of each source material are shown on Table 2. As expected, the DDA of the chitosans increased with increasing reaction time and temperature. Statistical analyses indicated that there were no batch differences, indicating that the de-acetylation process was consistent and reproducible for each source of chitosan material (Table 2). In this study, deacetylation was performed under nitrogen gas in an effort to prevent significant losses in MW so that the effects of DDA could be studied independently. In previous studies [14] nitrogen was not used and MW changed greatly as a result of the deacetylation reaction. However, performing the de-acetylation reaction under nitrogen failed to prevent significant loss in MW; the HS source material exhibited a maximum 37.4% decrease and the AT source material exhibited a maximum 63.0% decrease under the reaction conditions used. Tsaih and Chen [27] used a 50% NaOH solution to de-acetylate chitin at 99 °C or 140 °C for 1 to 9 h. Their results showed that the DDA of the resulting chitosan increased along with reaction time and/or reaction temperature while MW decreased. MW of those chitosans reacted at 140 °C were smaller than those at 99 °C. Cao *et al*. [11] also reported the MW of chitosan decreased 48.8% after one hour reaction with 40% NaOH at 100 °C. Therefore it appears that it is difficult to get chitosans with the same MW but different DDAs using this method. It is generally reported that MW is reduced by the increased processing necessary to obtain higher DDAs [28] and this appears to be the case in this study as well. Other methods such as re-acetylation reactions or performing reactions under pressure have been reported to be effective for controlling DDA without affecting MW and would be appropriate for further investigation [21,29].

Acid-base titration, potentiometer titration and UV-vis spectrophotometry were used to determine the DDA of deacetylated chitosans. Since the pH value of HS chitosan was 10.1, it was washed with deionized water to neutral pH before titration. Table 3 shows that DDAs determined by two titration methods were similar and the DDAs obtained from potentiometer titration and UV-vis were similar too, though the UV-vis method tended to give higher DDA values than either of the titration methods. While it is not clear why the UV-vis method would tend to yield higher DDA values than the titration methods, it is probably more important that the UV-vis method did provide comparable values. Past studies have also reported that titration methods yield DDA values comparable to FT-IR and H^1^-NMR methods suggesting that titration methods are useful and appropriate means for determining DDA [30,31]. Among the three methods used in this study, it is our opinion that the acid-base titration is the better method since it is convenient, easy, highly repeatable and does not require expensive equipment. These results also suggest that the method of DDA determination should be reported along with the DDA and MW of chitosans used in biomedical studies since values may vary with method.

The ash and protein contents of both sets of chitosans decreased with deacetylation processing times (Table 4). For HS, ash content significantly decreased from 3.22% to 0.85% and for AT, a 0.22% to 0.03% significant reduction was measured. The decrease in ash content with increasing DDA was attributed to extending NaOH reaction time and washing procedures. The protein assay results showed that residual protein content decreased from 1.06% to 0.86% for HS and from 0.84% to 0.56% for AT with processing to increase DDA. The reason for the decrease in protein was that when the chitosan materials were reacted with NaOH, NaOH also reacted with any residual protein, resulting in decreased protein levels. The decrease of ash and residual protein content increases the purity of chitosan. PBS and HAc were both used to prepare the standard protein solutions. The protein content determined was lower when using HAc than PBS since acid may alter protein structure. Since chitosan was dissolved in 1 wt% HAc, using HAc to prepare the protein standard is more reasonable.

**Table 4:** Protein and ash content of deacetylated chitosan.

Sample	Protein content % (in PBS)	Protein content % (in HAc)	Ash content %
HS0	1.55 ± 0.23	1.06 ± 0.37	3.215 ± 0.049
HS1	1.40 ± 0.20	1.05 ± 0.21	1.480 ± 0.084
HS2	1.45 ± 0.21	0.92 ± 0.17	1.239 ± 0.066
HS3	1.22 ± 0.10	0.87 ± 0.13	1.243 ± 0.144
HS4	1.21 ± 0.04	0.86 ± 0.22	0.847 ± 0.027
AT0	1.03 ± 0.09	0.84 ± 0.27	0.222 ± 0.010
AT1	1.16 ± 0.10	0.92 ± 0.39	0.055 ± 0.004
AT2	1.15 ± 0.16	0.94 ± 0.19	0.025 ± 0.004
AT3	1.07 ± 0.18	0.56 ± 0.06	0.051 ± 0.005

**Figure 1:**
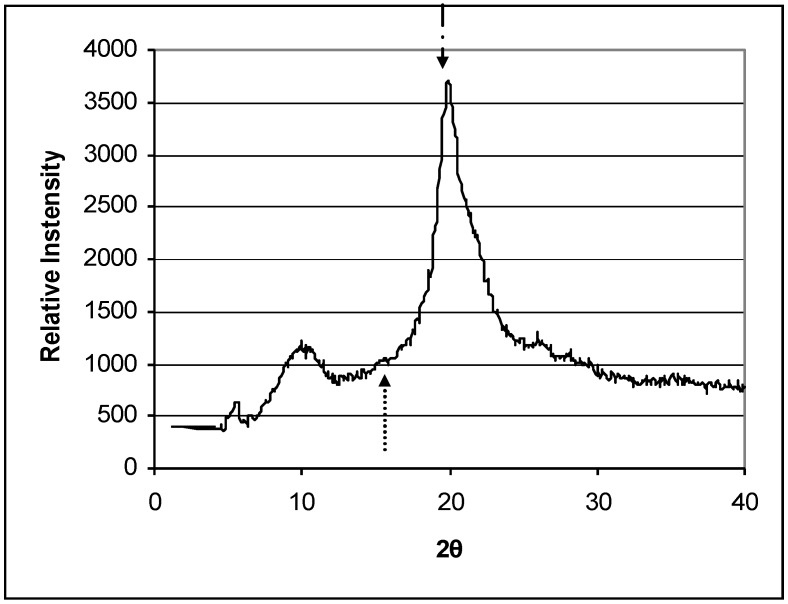
X-ray diffraction pattern. The HS0 72.6% DDA chitosan powder Diffractogram is representative of the other chitosan materials evaluated in this study. Peaks at 2θ ~ 10° and 20° are indicative of hydrated, partially crystalline materials. Arrows indicate amorphous background level and crystalline peaks used to calculate crystallinity index (amorphous background level: ↑ 2θ = 16°; crystalline peaks: ↓ 2θ = 20° for powder).

**Figure 2:**
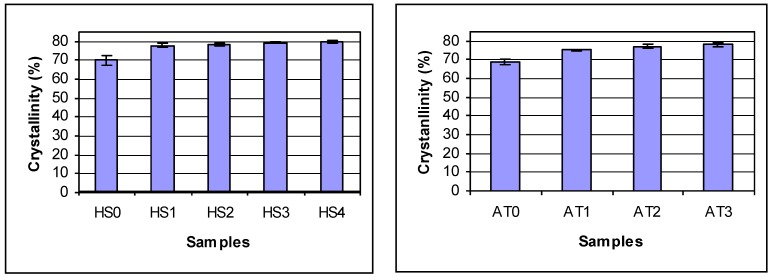
The crystallinities of deacetylated chitosans. Statistics analysis shows for AgraTech chiotsan: no difference in batches (p = 0.99) and differences between reactions (p = 0.001) by 2 Factor ANOVA. By Tukey’s HSD test: ATO is different from all others, AT1 and AT3 different, AT1 and AT2 are not different and AT2 and AT3 are not different; For HS chitosan, there are differences due to reaction time (p = 0.0001) by 2 Factor ANOVA. By Tukey’s HSD test: HS0 is different from all others. All others are the same.

**Figure 3:**
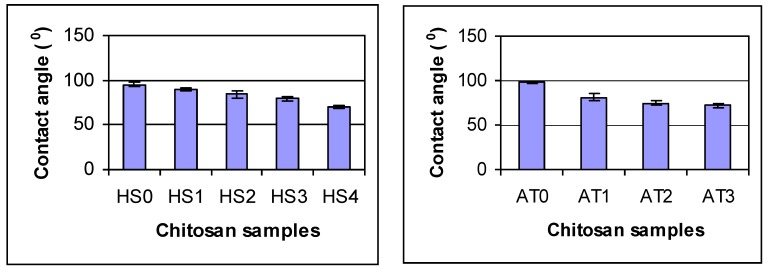
Contact angle of deacetylated chitosan. Statistics analysis shows by 2 Factor ANOVA: there are differences due to chitosan type (p = 0.0042) and processing time (p = 0.001). For Agratech chitosans: AT0 is different from all others, AT1 is different from all others, AT2 and AT3 are the same (p < 0.05). For HS chitosan: all groups are different except HS2 and HS3 are the same (p < 0.05). All comparisons made by Tukey’s HSD test.

In order to determine how chitosan DDA affects the properties of chitosan, the crystallinity, thermal stability and contact angle were measured. Figure 1 shows the X-ray diffraction pattern of chitosan powder. The crystallinities of the two series of deacetylated chitosans were shown in Figure 2. Chitosan crystallinity increased with increasing DDA. Jaworska *et al*. reported that the chitin was much more crystalline than deacetylated materials [6]. The degree of crystallinity depends on the deacetylation degree of the chitosan: the higher the DDA, the higher the degree of crystallinity [26]. This may be attributed to the fact that chains of chitosan with higher DDA are more flexible and have fewer large acetyl side groups. These are consistent with the results of the present study. In contrast, the contact angles of chitosans decreased with increasing DDA indicating that the hydrophilicity of chitosan increased with increasing DDA (Figure 3). The smaller the water contact, the better the surface wettability. The increase in wettability may be related to an increase in the number of free amino groups with increasing DDA. These amino groups may become protonated at neutral pH resulting in a high positive surface charge that promotes wettability [28,30].

The results of this study clearly show that processing conditions used to alter DDA affects many physiochemical properties of chitosan. Changing the DDA of chitosan also changed other important properties such as MW, ash content, protein content, and crystallinity. In previous studies, these complex effects were not well documented [14,15,21,22]. These changes could have a significant effect on the biological response to chitosan, and this was investigated in the second part of the study.

**Figure 4:**
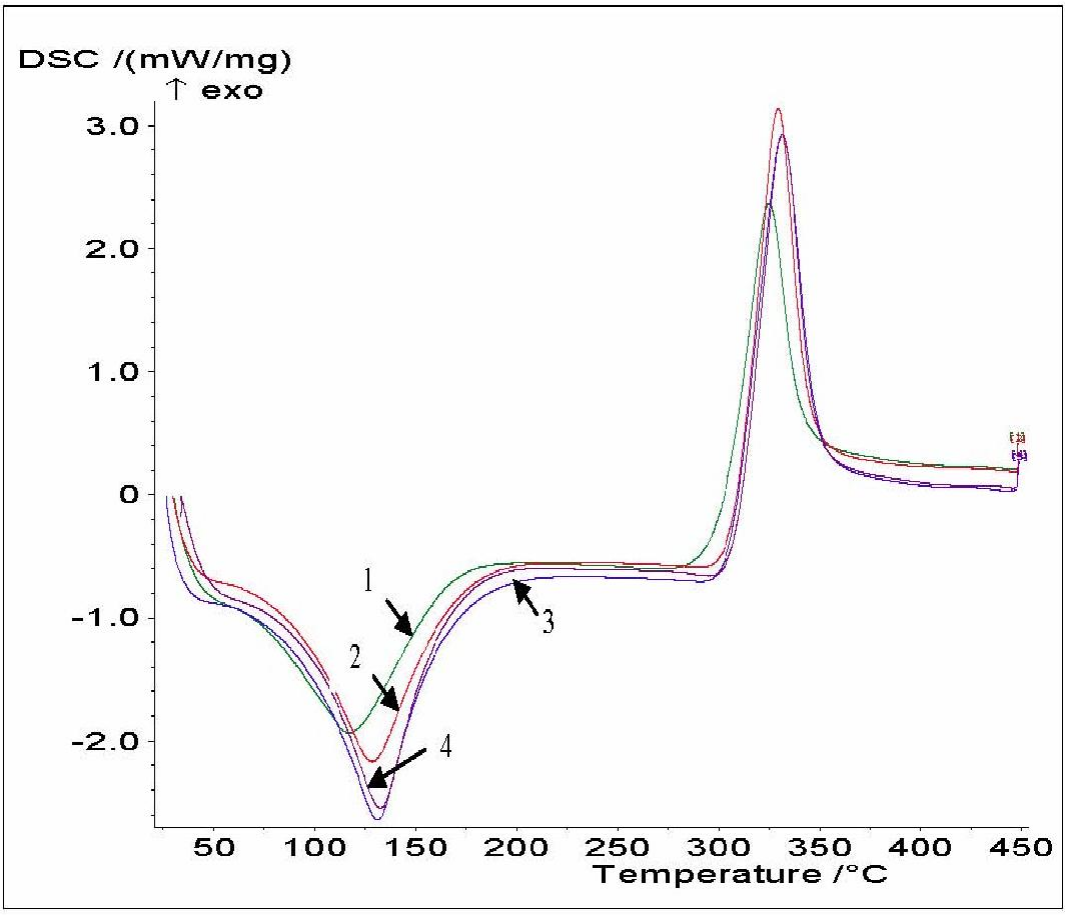
DSC thermograms of AT0 (**1**), AT1 (**2**), AT2 (**3**) and AT3 (**4**).

The examination of thermograms (Figure 4 and Table 5) reveals that there are differences in the endotherm peak and position, indicating that these chitosans differ in their water holding capacity and strength of water-chitosan interaction. All the chitosan samples had a wide endothermic peak centered between 120 and 140 °C with an onset at around 90 °C. The endothermic peak area (ΔH) increased with increasing chitosan DDA. The second thermal event was a wide exothermic peak, and its area was used to express the overall exothermic effect connected with decomposition, ΔH. Because the chitosans have similar structural characteristics, no remarkable differences in the exothermic transition were observed. The exothermic peak in chitosan AT0 at 209 °C was shifted to a higher temperature (252 °C) for chitosan AT3. The higher DDA and crystallinity of AT3 lead to increased molecular stability which could increase T_p_. ΔH and the decomposition peak temperature (T_p_) increased with increasing chitosan DDA. A similar behavior was observed for HS chitosans.

**Table 5:** Thermal transitions of chitosan decaetylated under different conditions (T_0_: onset temperature; T_p_: peak temperature; T_c_: completion temperature; ΔH: enthalpy).

	Endotherm			
Chitosan	To	Tp	Tc	ΔH J/g
AT0	77.9 ± 2.2	119.2 ± 2.3	167.6 ± 2.3	195.1 ± 11.5
AT1	90.4 ± 1.8	127.1 ± 2.7	166.0 ± 2.0	239.9 ± 8.5
AT2	96.2 ± 1.5	130.7 ± 0.7	163.0 ± 1.9	265.1 ± 23.3
AT3	99.0 ± 1.8	132.6 ± 0.2	163.1 ± 1.2	254.9 ± 7.2
HS0	84.0 ± 1.6	126.6 ± 0.9	173.5 ± 4.0	230.7 ± 8.0
HS1	86.6 ± 6.2	127.8 ± 3.2	168.8 ± 2.3	236.2 ± 0.7
HS2	89.2 ± 4.5	128.7 ± 1.9	165.8 ± 1.6	270.1 ± 2.1
HS3	88.2 ± 3.4	128.0 ± 2.3	162.9 ± 1.0	254.2 ± 4.9
HS4	96.7 ± 2.1	130.1 ± 0.7	160.7 ± 1.0	249.7 ± 5.7
	**Exotherm**			
**Chitosan**	**To**	**Tp**	**Tc**	**ΔH J/g**
AT0	301.6 ± 0.0	325.3 ± 0.3	340.1 ± 0.1	−208.9 ± 6.6
AT1	307.8 ± 0.4	329.8 ± 0.3	344.5 ± 0.4	−251.1± 7.6
AT2	306.3 ± 0.5	331.4 ± 0.5	348.5 ± 0.1	−276.4 ± 6.6
AT3	309.1 ± 0.4	332.0 ± 0.4	347.5 ± 0.3	−253.0 ± 2.8
HS0	306.3 ± 0.4	323.4 ± 0.4	338.4 ± 0.3	−172. 5 ± 5.6
HS1	305.9 ± 0.1	326.2 ± 0.1	340.0 ± 0.2	−216.8 ± 3.7
HS2	307.0 ± 0.4	328.0 ± 0.0	341.6 ± 0.4	−228.3 ± 1.8
HS3	307.0 ± 0.3	328.8 ± 0.1	342.6 ± 0.1	−231.9 ± 4.6
HS4	307.6 ± 0.7	331.1 ± 0.1	344.7 ± 0.1	−219.1± 0.7

Endotherm: for ΔH, by 2 Factor ANOVA: no differences due to chitosan type (p = 0.1418) and there are differences due to processing time (0.0007). For Agratech: AT0 is different from AT2 and AT3. No other differences detected (p < 0.05). For HS: HS0 is different from HS2, HS3 and HS4. HS1 and HS2 are different. HS2 and HS4 are different (p < 0.05).

For T_p_, by 2 Factor ANOVA: no differences due to chitosan type (p = 0.5605) and there are differences due to processing time (p = 0.0404). For Agratech: AT0 is different from all others. No other differences detected (p < 0.05). For HS: no differences in any groups.

Exotherm: for ΔH, by 2 Factor ANOVA: there are differences due to chitosan type (p = 0.0001) and processing time (p = 0.0001). For Agratech: AT0 is different from all others and no other differences detected (p < 0.05). For HS: HS0 is different from all others and no other differences detected (p < 0.05).

For T_p_, by 2 Factor ANOVA: there are differences due to chitosan type (p = 0.0001) and processing time (p = 0.0001). For Agratech: AT0 is different from all others. AT1 is different from AT3 (p < 0.05). For HS: all groups are different (p < 0.05). All groups compared by Tukey’s HSD test.

**Figure 5:**
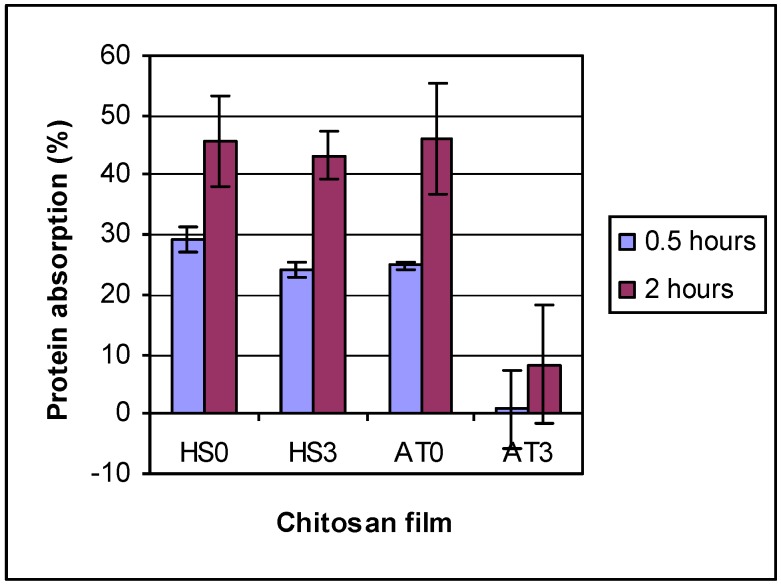
The protein absorption on chitosan films. 2 Factor ANOVA showed differences due to chitosan type and time. AT3 and control were different from all others, but were not different from each other. 2 h also differs from 0.5 h.

To determine the effects of DDA and other physiochemical properties on the biological performance of chitosan, protein adsorption on different DDA chitosan films was conducted at two time points (0.5 and 2.0 h) in 500 μg/mL albumin in PBS solution. The results (Figure 5) showed that protein adsorption was between 20% to 30% on HS chitosans and AT0, but for AT3 only 0.8% after 0.5 h. The protein adsorption increased with absorption time to between 40% to 48% (after 2 h) on HS chitosans and AT0, but only 8.3% for AT3. Also it was found that protein absorption decreased with increasing chitosan DDA. Similar results for changes in hydrophilicity and protein adsorption with DDA have been reported [11,32]. However, because processing conditions to change DDA also changed many other physiochemical properties of the chitosan, it is not possible to determine if the increased protein adsorption observed was directly related to the increase in DDA of the chitosan or to some other factor, such as changes in ash or protein content or MW. Higher DDA chitosan was more pure as evidenced by lower ash and protein contents in the higher DDA chitosans, and this could be responsible for some of the increased protein absorption to higher DDA chitosan films. This is something that should be further investigated in future studies to try and determine if DDA actually affects protein absorption independently of other factors. To do this, however, it will be necessary to use other methods of deacetylating chitosan without changing other properties such as MW, ash content, or protein content which may be more complicated than the process used in this study.

**Figure 6:**
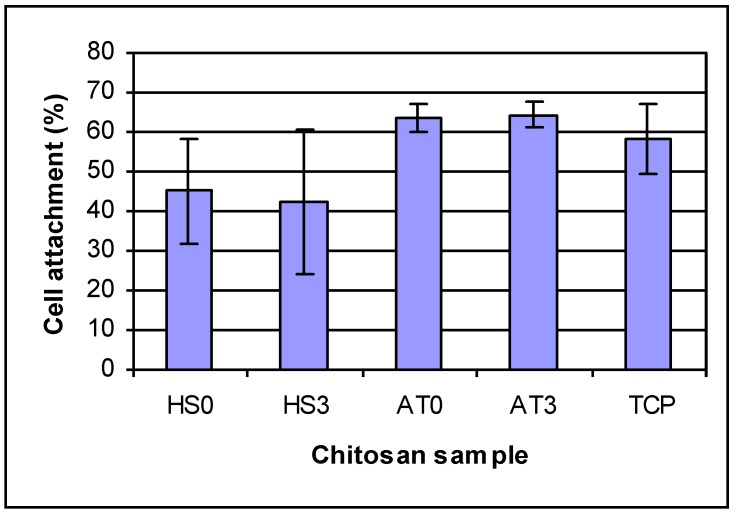
HEPM cell attachment on chitosan films for 4 h. For cell attachment: there are differences by ANOVA Groups that are the same: (1) HS0 and HS3; (2) HS0 and TCP; (3) TCP, AT0 and AT3. AT0 and AT3 are both significantly different from HS0 and HS3.

The cell attachment results are shown in Figure 6. After 4 h, HEPM cell percent attachments were 45.21% and 42.15% for chitosan AT0 and AT3; 63.26% and 64.27% for chitosan AT0 and AT3; 58.26% for tissue culture plate, respectively. Different chitosan films have different attachment percentage, but there were no differences for the effect of different DDAs of same kind chitosan on cell attachment. 

**Figure 7:**
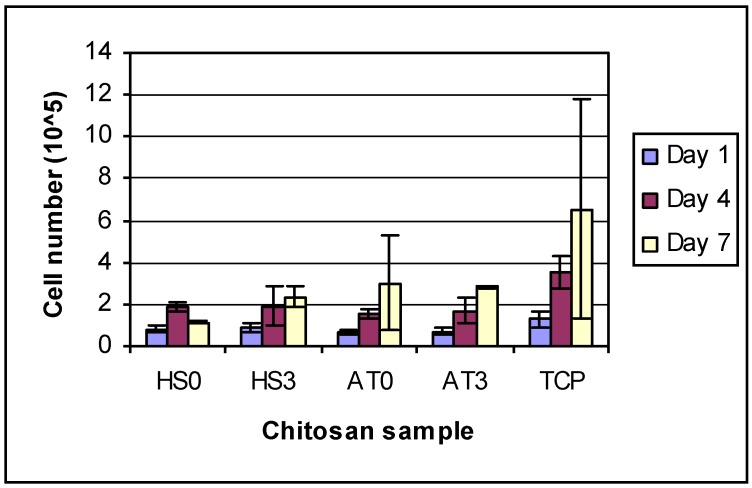
HEPM cell proliferation number on day 1, 4 and 7. Overall 2-way ANOVA: There are differences between days and between groups. TCP was significantly different from all other groups. Each day was significantly different from other days.

The initial seeded cell concentration was 1 × 10^5^ cell/cm^2^. From the results of cell proliferation (Figure 7), it was observed that the concentration of cells on all chitosan films was less than the initial seeded concentration after 1 day culture. The reason may be that some cells went into the swollen chitosan films. The cell numbers for different DDAs of same kind of chitosan were almost the same after one day of growth, but by day 4 and day 7, the cells on higher DDA chitosan films had proliferated more than those on low DDA chitosan films. Recently, Amaral *et al*. [33] investigated the short-term proliferation of MG-63 osteoblasts on different DDA (from 51% to 96%) chitosan films. Their results mirrored the results of this study in that cell growth was higher on films with the highest DDA (96%) as compared to cells on films with lower DDAs. However, it is important to note that it is impossible to determine if DDA directly affected cell proliferation or if it was due to other physiochemical changes in the chitosan that resulted from the deacetylation process. Amaral, *et al*. also showed that the MW of chitosan decreased with increasing DDA [33], although any changes in other physicochemical properties were not reported in that study.

Although other studies have reported some information on the biological properties of various DDA chitosans, this study is significant in that it provides a comprehensive analysis of the effects of the deacetylation process from two different source materials on the physiochemical and biological properties of chitosan. In the past, research on the effect of chitosan DDA on biological response on cell proliferation has been contradictory and largely dependent on manufacturer reported chitosan characteristics. Some studies indicate that cell proliferation increases with DD A [10,11], while others show that there is no discernable effect [20]. These apparently contradictory results may be due in part to other factors as identified in this work, such as such as protein and ash content and MW which are influenced by processing and which may also play a significant role in the biological response to chitosan. Protein and ash content and MW can also vary widely for different source materials of chitosan, so if different sources of chitosan are used, these confounding factors may become significant. More studies are needed to further examine the effects of each of these properties of chitosan independent of changes in DDA.

The original purpose of this study was to determine how DDA affected chitosan physiochemical and biological properties. However, changing the DDA changed many other properties of the chitosan, such as MW and protein and ash content as well. . Hence it is unclear if physiochemical and biological properties were dependent on any of these properties alone or by some unidentified interaction among them. Future studies are needed in which chitosans with the same MW but different DDA or with the same DDA but different MW are evaluated to unambiguously determine the influence of DDA or MW on *in vitro* and *in vivo* performance.

## 5. Conclusions

Two kinds of chitosans with low DDA were deacetylated using 45% NaOH under an inert atmosphere to get two series of chitosans with increasing DDAs. While the process was repeatable, the molecular weight decreased with increasing deacetylation reaction time and temperature. The deacetylation reaction also decreased the protein and ash content of all chitosan materials. The crystallinity increased and the contact angle decreased with increasing chitosan DDA. The chitosan DDA also changed the decomposition temperature, which increased with increasing chitosan DDA in spite of the decreased MW. The protein adsorption decreased and bone cell growth increased with the increase of chitosan DDA. Processing conditions increased DDA and influenced physicochemical and biological properties. However, additional studies are needed to unambiguously determine the influence of DDA or MW on *in vitro* and *in vivo* performance of chitosan materials.

## References

[1] Di Martino A., Sittinger M., Risbud M.V. (2005). Chitosan: A versatile biopolymer for orthopaedic tissue-engineering. Biomaterials.

[2] Senel S., McClure S.J. (2004). Potential applications of chitosan in veterinary medicine. Adv. Drug Deliv. Rev..

[3] Khor E., Lim L.Y. (2003). Implantable applications of chitin and chitosan. Biomaterials.

[4] Bumgardner J.D., Wiser R., Gerard P.D., Bergin P., Chestnutt B., Marini M., Ramsey V., Elder S.H., Gilbert J.A. (2003). Chitosan: Potential use as a bioactive coating for othopaedic and craniofacial/dental implants. J. Biomater. Sci. Polym. Ed..

[5] Kumar M.N.V.R. (2000). A review of chitin and chitosan applications. React. Funct. Polym..

[6] Jaworska M., Sakurai K., Gaudon P., Guibal E. (2003). Influence of chitosan characteristics on polymer properties: I: Crystallographic properties. Polym. Int..

[7] Freier T., Koh H.S., Kazazian K., Shoichet M.S. (2005). Controlling cell adhesion and degradation of chitosan films by N-acetylation. Biomaterials.

[8] Harish Prashanth K.K., Kittur F.S., Tharanathan R.N. (2002). Solid state structure of chitosan prepared under different N-deacetylating conditions. Carbohydr. Polym..

[9] Prasitsilp M., Jenwithisuk R., Kongsuwan K., Damrongchai N., Watts P. (2000). Cellular responses to chitosan *in vitro*: The importance of deacetylation. J. Mater. Sci. Mater. Med..

[10] Hidaka Y., Ito M., Mori K., Yagasaki H., Kafrawy A.H. (1999). Histopathological and immunohistochemical studied of membranes of deacetylated chitin derivatives implanted over rat calvaria. J. Biomed. Mater. Res..

[11] Cao W.L., Jing D.H., Li J.M., Gong Y.D., Zhao N.M., Zhang X.F. (2005). Effects of the degree of deacetylation on the physicochemical properties and Schwann cell affinity of chitosan films. J. Biomater. Appl..

[12] Tsaih M.L., Chen R.H. (2003). The effect of reaction time and temperature during heterogenous alkali deacetylation on degree of deacetylation and molecular weight of resulting chitosan. J. Appl. Polym. Sci..

[13] Ogawa K., Yui T. (1993). Structure and function of chitosan. 3. Crystallinity of partially N-acetylated chitosans. Biosci. Biotech. Bioch..

[14] Zhang Z.T., Chen D.H., Chen L. (2002). Preparation of two different serials of chitosan. J. Dong Hua Univ. (Eng. Ed.).

[15] Knaul J.Z., Kasaai M.R., Bui V.T., Creber K.A.M. (1998). Characterization of deacetylated chitosan and chitosan molecular weight review. Can. J. Chem..

[16] Xu Y., Du Y. (2003). Effect of molecular structure of chitosan on protein delivery properties of chitosan nanoparticles. Int. J. Pharm..

[17] Zhang H., Neau S.H. (2001). *In vitro* degradation of chitosan by a commercial enzyme preparation: effect of molecular weight and degree of deacetylation. Biomaterials.

[18] Nunthanid J., Puttipipatkhachorn S., Yamamoto K., Peck G.E. (2001). Physical properties and molecular behavior of chitosan films. Drug Dev. Ind. Pharm..

[19] Hamilton V., Yuan Y., Rigney D.A., Chesnutt B.M., Puckett A.D., Ong J.L., Yang Y., Haggard W.O., Elder S.H., Bumgardner J.D. (2007). Bone cell attachment and growth on well-characterized chitosan films. Polym. Int..

[20] Chatelet C., Damour O., Domard A. (2001). Influence of the degree of acetylation on some biological properties of chitosan films. Biomaterials.

[21] Huang M., Khor E., Lim L.Y. (2004). Uptake and Cytotoxicity of Chitosan Molecules and Nanoparticles: Effects of Molecular Weight and Degree of Deacetylation. Pharm. Res..

[22] Gao Q., Wan A., Zhang Y. (2007). Effect of Reacetylation and Degradation on the Chemical and Crystal Structures of Chitosan. J. Appl. Poylm. Sci..

[23] Jiang T.D. (2001). Chitosan.

[24] Broussignac P. (1970). Un polymere natural pecu cannu dans 1’ industrie e chitosane. Chim. Ind.-Genie Chim..

[25] Muzzarelli R.A.A., Rocchetti R. (1985). Determination of the degree of acetylation of chitosans by first derivative ultraviolet spectrophotometry. Carbohydr. Polym..

[26] Dunn Q., Li E.T., Grandmaison E.W., Goosen M.F.A., Goosen M.F.A. (1997). Applications and Properties of Chitosan. Applications of Chitin and Chitosan.

[27] Urbanczyk G.W., Lippsymonowicz B. (1994). The influence of processing terms of chitosan membranes made of differently deacetylated chitin on the crystalline-structure of membranes. J. Appl. Polym. Sci..

[28] Gong H.P., Zhong Y.H., Li J.C., Gong Y.D., Zhao N.M., Zhang X.F. (2000). Studies on nerve cell affinity of chitosan-derived materials. J. Biomed. Mater. Res..

[29] Abdou E.S., Nagy K.S.A., Elsabee M.Z. (2008). Extraction and characterizatin of chitin and chitosan from local sources. Bioresour. Technol..

[30] Chellat F., Tabrizian M., Dumitriu S., Chornet E., Rivard C.H., Yahia L. (2000). Study of biodegradation behavior of chitosan-xanthan microspheres in simulated physiological media. J. Biomed. Mater. Res..

[31] Domszy J.G., Roberts G.A.F. (1985). Evaluation of infrared spectroscopic techniques for analyzing chitosan. Makromol. Chem..

[32] Tomihata K., Ikada Y. (1997). *In vitro* and *in vivo* degradation of films of chitin and its deacetylated derivatives. Biomaterials.

[33] Amaral I.F., Cordeiro A.L., Sampaio P., Barbosa M.A. (2007). Attachment, spreading and short-term proliferation of human osteoblastic cells cultured on chitosan films with different degrees of acetylation. J. Biomater. Sci. Polym. Ed..

